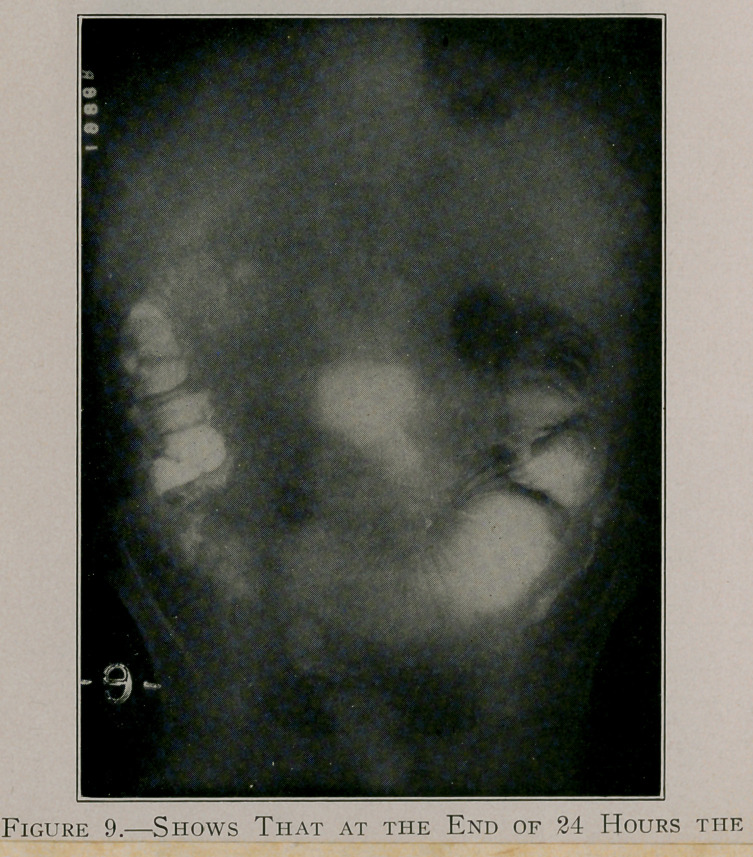# Appendicitis

**Published:** 1914-04

**Authors:** James A. Mac Leod, Frederick B. Bowman

**Affiliations:** Buffalo, N. Y.; Buffalo, N. Y.


### BUFFALO MEDICAL JOURNAL

VOLUME 69 APRIL, 1914 No. 9

### ORIGINAL ARTICLES

*The right is reserved to decline papers not dealing with practical medical and surgical subjects and such as might offend or fail to interest readers. Contributors are solely responsible for opinions, methods of expression and revision of proof.*

### Appendicitis

BY JAMES A. MAC LEOD, M.D., M.R.C.S., Eng.
Buffalo, N. Y.
and
FREDERICK B. BOWMAN, M.D.
Buffalo, N. Y.

THE subject of appendicitis has been for many years one of endless discussion and voluminous writing. It is considered by many of the profession as a disease easy of diagnosis and simple of treatment. It is, however, one in which the errors in diagnosis are exceedingly numerous and in which the greatest skill may be required in its treatment. In this paper we shall discuss the pathology, diagnosis and treatment in a more or less general way and shall make use of the summaries of some of our cases in illustrating various points at issue.

The pathology has been discussed under many heads, but the following divisions will serve as a basis for the description of the morbid condition.

1. Acute Catarrhal Appendicitis.

2. Acute Suppurative Appendicitis.

3. Obliterative Appendicitis.

4. Chronic Appendicitis.

In the acute catarrhal condition the organ, externally, may appear quite normal, with the exception of, possibly, some injection of the blood vessels. On section the mucosa is boggy and somewhat thickened and perhaps discolored. Feces may or may not be present. Microscopically the serosa may appear quite normal and the muscular layer will probably show nothing pathological. There may be some cellular infiltration into the submucosa and the vessels here will be engorged. The mucosa itself is distorted, the glandular cells swollen, granular, poorly stained and irregular. The lymphoid tissue will be found more or less hyperplastic. There may be all grades in this catarrhal condition ranging from only a slight oedema of the mucosa to almost a necrotic process.

In acute suppurative appendicitis the causative factor is bacterial. The serosa is sandy looking and possibly small threads of fibrinous material may be present. The blood-vessels stand out prominently. The whole organ may appear red and swollen. On section the wall may be thick or thin, depending on the time of infection, and the lumen patent and perhaps containing an oval fecolith, which has many times been mistaken for a date seed. Microscopically, the serosa is seen to be thickened, and just beneath are numerous polynuclear neutrophiles and often a large percentage of eosinophiles. The blood vessels throughout the wall are engorged. Infiltrating all of the layers will be found polynuclear leucocytes in more or less abundance, and all the cells concerned in any acute inflammatory condition, and bacteria also may be stained. The mucosa is very irregular and in certain areas may have dissappeared altogether or may be found lying in the lumen in a necrotic condition, surrounded by leucocytes and other cells and masses of bacteria. If ulceration has taken place it is simply a later stage in the suppurative process. A section from such an ulcer shows a complete lack of any anatomical structure and the normal tissues present replaced by bacteria, leucocytes, fragmented nuclei and all the constituents of an extreme inflammatory process. If healing has begun early granulation tissue will be found.

In obliterative appendicitis a somewhat similar picture may be presented, though in this condition the immediate etiology is distinctly different. The remote etiological factor causing the condition may have been catarrhal with a clearing up of the inflammatory picture, but with the formation each time of more or less scar tissue. This finally has cut off the single blood supply and gangrene has resulted. Any process either in the appendix or in its immediate vicinity causing the compression of the arterial supply would have the same effect. The organ appears dark brownish red usually, is bad smelling and covered with greyish pasty looking exudate. On microscopical examination it is seen that there is no normal anatomical picture left. When gangrene occurs the surrounding tissues quickly throw out an exudate of fibrin, and becoming matted together wall off the general peritoneal cavity. These adhesions early in the process are quite fragile and easily torn, but if allowed to remain they may become fibrous.

In chronic appendicitis, the acute or catarrhal condition occurring from time to time causes the formation of fibrous tissue which may not cut off the blood supply, and instead of the organ becoming gangrenous it becomes a fibrous cord.

All grades of the above conditions may be found merging one into the other, but these are mentioned as applying directly to the subsequent discussion of the subject.

### The Diagnosis of Acute Appendicitis.

In the diagnosis we have to differentiate appendicitis from all the acute disturbances of the abdominal cavity, and the following conditions have, in our experience, been the source of the most difficulty:

.1 Acute pyelitis.

2. Acute cholecystitis.

3. Acute salpingitis and acute ovaritis.

4. Typhoid fever.

5. The rupture of an extra-uterine pregnancy.

6. The rupture of a gastric or duodenal ulcer.

The classical signs and symptoms, as we all know, are the acute onset—pain, elevated temperature, increased pulse rate, nausea and vomiting, constipation and spasm of the muscles on the right side of the abdomen. An examination of the blood shows an increase in the number of the leucocytes with a preponderance of the polynuclear cells. The diagnostic value of the blood count depends more on the polynuclear percentage than on the actual count of the leucocytes; for example, a high leucocyte count with a low polynuclear percentage is a much more favorable sign than a moderate leucocytosis with a high polynuclear percentage. In favorable cases the signs and symptoms may subside in a few hours, resolution occurring. It is sometimes noted in the very acute cases that there is a sudden cessation of the pain with a distinct improvement in the clinical picture. This may be due to two causes. First: The distended and inflamed appendix may empty itself into the cæcum, and this is quickly followed by resolution of the attack. Second: The distended and gangrenous appendix may rupture and empty itself into the general peritoneal cavity; this is very shortly followed by a rapidly spreading general peritonitis. As stated above, this phenomenon occurs in the very acute cases, and, therefore, it is most important not to be misled by any apparent improvement. If the signs and symptoms do not improve within the first twenty-four hours, it is probable that the disease, instead of resolving, will become more serious, the signs and symptoms becoming more marked.

In the more serious cases the appendix becomes gangrenous, perforates and causes a rapidly spreading general peritonitis. In those cases running a less acute course adhesions are formed, shutting off the general peritoneal cavity from the focus of the infection, and we have the formation of an abscess, in which the appendix is generally found to be gangrenous, and sometimes lying detached in the abscess cavity.

If the case is seen early and the signs and symptoms are typical, the diagnosis may be an easy one, but the clinical picture is only too often deficient and in some cases very puzzling. There is no more pernicious thing for a man to do than to make the diagnosis of appendicitis, because he finds a patient suffering from an acute pain in the right abdomen; this, however, is very commonly done.

1. In acute pyelitis there is the history of an acute onset, pain in the right side, elevated pulse rate and temperature; nausea and vomiting may or may not be present; the muscles on the right side are not as a rule rigid; colonic irritation and abdominal distension are usually present; the blood count is high with a preponderance of the polynuclear cells. The pain may be severe, but, on examination, it is found to be more pronounced in the costo-vertebral angle of the right side. Examination of the urine shows pus cells, casts, possibly blood cells and bacteria in great numbers. It is, therefore, most important in all cases of acute abdominal disease to investigate the urine and the presence or absence of pain in the costo-vertebral angles As an illustration of this we give a summary of the case of Mr. S. We were asked to operate for acute appendicitis. He had been ill for three days. He had been nauseated and had vomited twice. His bowels had been moved by cathartics. His temperature was 104.5 and his pulse rate was 100. Examination of the abdomen revealed that it was markedly distended, due largely to colonic distension; the abdominal muscles were not rigid; there was some tenderness over the cæcum, ascending and transverse colon; there was marked tenderness at the right costo-vertebral angle, and on deep palpation over the right kidney. A single sample of urine was taken and found to contain pus, blood cells, casts and bacteria in great numbers. Cultures from the urine showed the presence of streptococci. Under vaccine and medicinal treatment he made an uneventful recovery.

2. In acute cholecystitis the signs and symptoms are very similar to those of acute appendicitis with pain, temperature, etc. There is, however, very commonly associated with it a more or less marked jaundice, and the appearance of bile salts in the urine. The pain is usually more pronounced in the right upper quadrant and radiates through to the back below the scapula; it is elicited most markedly by the hammer stroke test of Murphy. The absence of jaundice and bile salts in the urine do not, however, exclude gall bladder disease, as these are only present when there is an associated inflammation of the common duct. In their absence the hammer stroke test is of great importance. There may or may not be an associated enlargement of the liver. If the muscular rigidity is not too marked the gall bladder may be palpated and found to be enlarged and very sensitive. If the muscles are too rigid to permit palpation of the organ percussion may demonstrate the enlargement, but the enlargement may be hidden by the associated distension of the colon, which in our experience is very commonly present.

3. In acute salpingitis and ovaritis the signs and symptoms are very similar to those of acute appendicitis, but they are more pronounced in the lower abdomen. There is the presence or history of an acute vaginitis. A pelvic examination demonstrates that the uterus is fixed with a very sensitive swelling at one side of it. The complement-fixation test for gonorrhœa may be positive where the causative factor is the gonococcus. There is a well marked leucocytosis with a preponderance of the polynuclear cells. Examination of the urine may or may not show pus cells, bladder cells and bacteria, and this depends upon whether or not there has been an acute urethritis associated with the vaginitis.

4. In typhoid fever the onset is insidious, and very often the patient, though feeling below par, keeps along the even tenor of his way for about a week before calling in his physician. At this period of the disease he may feel nauseated and may even vomit. There is usually some distension of the abdomen; pain in the right side may be present; gurgling may usually be obtained by palpation over the right iliac fossa. Blood examination usually shows a leukopenia, but where there is a secondary bacterial infection of the ulcers we may find a leucocytosis with a preponderance of the polynuclear cells. A Widal reaction may be negative, in fact, it may not be obtained until the end of the second week of the typhoid; a blood culture is, however, usually positive after the second or third day. Diarrhœa or constipation may be present. The abdominal muscles, as a rule, are not rigid, but there may be considerable abdominal distension. Rose red spots are not evident and the spleen is not much enlarged. Later on in the course of the disease the clinical picture and Widal reaction, where they have been heretofore vague, become typical and demonstrate the nature of the trouble.

As an illustration of this we give a summary of the case of Mr. M. When first seen by us Mr. M. had been confined to his bed for twenty-four hours. During that time a careful laboratory investigation had been carried out. The blood showed a marked leucocytosis with a preponderance of the polynuclear cells. A Widal reaction had been negative. Examination of the urine had been negative. On examination we found moderate distension with some tightening of the abdominal muscles. There was some tenderness on palpation over the cæcum, and this extended over the ascending and transverse colon; there was some gurgling on palpation over the cæcum; there was no tenderness over the back and none over the gall bladder. The spleen was not enlarged; there were no rose red spots. There was no nausea and the bowels were constipated. He gave a history of feeling "seedy" for some time. He had suffered from indigestion and constipation for years. No diagnosis was made and the laboratory investigation was continued. The clinical picture continued much the same with a marked leucocytosis. On the fourth day after our first consultation the Widal reaction was positive and he had a severe intestinal hemorrhage. During the course of the disease he had frequent intestinal hemorrhages but eventually made an excellent recovery. From the presence of the leucocytosis, instead of the customary leukopenia, and the frequency of the intestinal bleeding he undoubtedly had a severe secondary infection of the typhoid ulcers.

5. In ruptured extra-uterine pregnancy the onset is sudden and accompanied by shock, which varies according to the amount of the hemorrhage. There is the history of a missed period or two; there is intense pain in the lower quadrants of the abdomen; the face has an anxious expression; the temperature is subnormal and the pulse rate is much increased. Pelvic examination reveals a mass to one or the other side of the uterus. The patient is usually in too serious a condition to warrant any laboratory investigation and immediate operation is demanded. If, perchance, a diagnosis of acute appendicitis be made and operation performed a normal appendix and free blood in the abdomen quickly settles the question.

6. In perforation of a gastric or duodenal ulcer the onset is sudden with a sharp shooting pain radiating through to the back from the region involved. Shock is a prominent feature; the abdomen is distended; the liver dulness may be diminished. There is a history of previous gastro-intestinal disturbance. The temperature is not raised and where the shock is intense it may be subnormal; the pulse rate is increased. The condition of the patient is so serious that laboratory investigation is not warranted and abdominal section is imperative. On opening the abdomen free gas quickly settles the diagnosis.

In our experience the above discussed conditions are the important ones to be considered, but, as we have stated above, it is well to bear in mind that any acute abdominal disturbance may simulate or be simulated by acute appendicitis. For example: Acute pancreatitis, strangulated hernia, intestinal obstruction, intussusception and vovulus, movable kidney with torsion of its pedicle, ovarian cyst with torsion of its pedicle, and abdominal manifestations of erythema nodosum. An acute lesion arising outside of the abdominal cavity, for example a pneumonia, by having pain referred to the right side of the abdomen may stimulate appendicitis.

Where the case is not seen until an abscess has formed the signs, symptoms and blood picture are similar to those observed in the earlier stages of the disease, but they are not so marked. The temperature and pulse are those of a septic state and are governed, as is also the blood count, by the thickness of the limiting wall of adhesions. Where the wall is thick absorption is much lessened from the focus of infection and consequently the constitutional signs are not so active as where the limiting wall is thin, allowing free absorption. There is a well marked tumor formation in the right abdominal cavity and this may extend in any direction. The abdominal distension may be much lessened. The pain is limited to the area of the tumor. The nausea and vomiting will have disappeared. There may be lowering of the hemoglobin percentage on account of secondary anæmia.

In the diagnosis of appendicitis abscess the following are the important conditions to be differentiated:

1. Pelvic abscess.

2. Pelvic hematoma following the rupture of an extra-uterine pregnancy.

3. Perinephritic abscess and pyonephrosis.

In pelvic abscess the signs and symptoms of a septic process are present, but, in addition, there is the history of an acute pelvic inflammation. There is fixation of the uterus, which is pushed over to one side of the pelvis, and there is a swelling at one side, which causes a bulging down of the vaginal vault. The complement-fixation test is positive where the causative factor has been the gonococcus, and the importance of this reaction must not be underestimated.

In pelvic hæmatoma following the rupture of an extra-uterine pregnancy a tumor is present similar in character to that caused by a pelvic abscess, but it may extend high up into the abdomen. The presence or the history of a blood stained uterine discharge is important; and this may be slight in amount or copious, in fact, so copious as to endanger the life of the patient. If it be present a scraping from the endometrium may show typical decidual cells. Blood examination will show a marked lowering of the hæmoglobin percentage. Following a bleeding, and this may be small and repeated from time to time, there may be a leucocytosis, due perhaps to the absorption of fibrin ferment; this leucocytosis, however, does not rise so high as that occurring in an acute inflammatory process, and, moreover, it quickly subsides. If a secondary infection of the clot occurs, and this commonly happens, the signs become those of a true pelvic abscess. The Abderhalden test for pregnancy may be employed as an aid to a correct diagnosis.

As an illustration of this we give a summary of the case of Mrs. X. We were asked to see the patient, the diagnosis being intestinal obstruction arising in the course of an attack of typhoid fever. She had been ill three weeks. The chart showed an intermittent temperature. Two Widal reactions had been negative, no further laboratory investigation had been made. Examination of the abdomen revealed a large swelling occupying the right and lower quadrants of the abdomen. A diagnosis of abdominal abscess, probably of appendicitic origin, was made. On opening the abdomen a few hours later a dark brown fluid of offensive odor gushed out. It was decided not to proceed any further at that time but to have a full laboratory investigation instituted at once. On inquiring more fully into the history of the case it was ascertained that she had had during the entire course of the disease an irregular blood-stained discharge from the uterus. Laboratory investigation revealed that the fluid contained pus, blood cells in all stages of disintegration, bacillus colon and bacillus pyoscyaneous. Blood examination showed a marked leucocytosis with a preponderance of the polynuclear cells, and a low hemoglobin percentage. Examination of the uterine discharge showed typical decidual cells. A diagnosis of extra-uterine pregnancy with a secondary infection of the blood clot was made, and operation proved such to be the case.

3. In perinephritic abscess and pyonephrosis the tumor formation is more marked in the posterior region of the abdominal cavity; the cæcum and colon are pushed forwards and to the center, and are resonant over it. Blood examination, temperature and pulse are those of a septic state. Examination of the urine usually shows it to contain blood cells, pus cells and bacteria. It is, however, quite possible to have a nephritic abscess with no urinary signs; this may happen where the abscess has become walled off from the surrounding kidney tissue. In addition to the above discussed conditions the following should be considered in the differential diagnosis: Abdominal tumors, innocent and malignant, tubercular lesions of the abdomen, subphrenic and hepatic abscesses.

In the diagnosis of chronic appendicitis, as in the diagnosis of the acute condition, the problem on the one hand may be comparatively a simple one, where there is a distinct history of one or more acute attacks, and on the other hand it may be very puzzling and only arrived at by the exclusion of other chronic abdominal conditions. The disease may simulate or be simulated by many of the chronic disturbances. In a typical case there is the history of one or more acute attacks. There may be localized tenderness and a tendency to some tightening of the abdominal muscles on the right side. Constipation and flatuency may be prominent symptoms. Blood examination is not likely to be of much help. The temperature and pulse are not as a rule much affected. Where tenderness does not seem to be present, deep massage in the right iliac fossa may elicit it.

Where the signs and symptoms are not typical the problem is often a very puzzling one. We shall not discuss the differential diagnosis in detail from other chronic abdominal disorders, but shall treat the subject in a more or less general way. We shall give a brief summary of certain of our cases as illustrations of the points at issue. As in the diagnosis of the acute forms of appendicitis the laboratory is of great importance in aiding us to arrive at a correct diagnosis in the study of chronic cases. A thorough X-ray investigation is also essential in a large percentage of the cases. Some of the most puzzling cases are those usually referred to the internist with a complaint of various neuroses or disturbances of the gastro-intestinal tract. Study of these cases demonstrates that the underlying factor in their causation is motor derangement due to reflexes arising in the neighborhood of the appendix.

In chronic appendicitis adhesions are formed which, by their location or by fixing the appendix in some abnormal position, may give rise to remote reflex symptoms. In the case of a mobile cæcum the appendix may occupy many different positions. Adventitious membranes in the neighborhood of the ileo-cæcal valve may give rise to obstruction at that point in a manner similar to that caused by adhesions.

In the case of a retro-cæcal appendix adhesions to the sheath of the ileo-psoas muscle may be provocative of pain or distress on walking. As an illustration of this we give a summary of the case of Mr. V.: The patient in the year 1907 had three attacks of acute appendicitis in the course of three months; these were brief in duration and quickly ended in resolution. Soon after the third attack he noticed that on walking quickly a dull ache arose in the right side and radiated down into the thigh, and that this was only relieved by lying down. No other acute attack arose, but in the course of a few months we removed the appendix, which was discovered retro-cæcal and retro-peritoneal, lying adherent to the posterior wall of the cæcum and the anterior sheath of the psoas muscle. In retro-cæcal appendicitis it is possible, where a low grade infection is present, to have an abscess formed without it being detected as such. As an illustration of this we give the summary of the case of Mr. B.: The patient had been suffering from a general disturbance of the gastro-intestinal tract for about a year, during which time he had steadily lost in weight, about 30 pounds. There was no history of any acute attack of abdominal trouble. Examination revealed a swelling in the right side apparently continuous with the right kidney. There was no tenderness in the costo-vertebral angle. The cæcum covered it in front and was resonant over it. Blood examination showed a leucocytosis of 12,000 with 90 per cent. of polynuclear cells. Examination of the urine was negative. On account of the fact that most of the symptoms were confined to the gastro-intestinal tract and the absence of urinary signs the diagnosis of post cæcal abscess was made and operation revealed such to be the case.

In female patients the appendix may be adherent to the right ovary or tube. In such cases there are the associated signs and symptoms of chronic ovaritis or salpingitis or both, which, with those of the appendicitis are accentuated at the time of menstruation. The diagnosis here is very important on account of a possible future pregnancy. In the event of the patient becoming pregnant the stretching of the adhesions, as the pregnancy progresses, may lead to an interference with the blood supply of the appendix and excite an acute attack of appendicitis, which would prove a serious complication to the pregnancy.

Where a pelvic examination in suspected chronic appendicitis reveals a swelling to the right side of the uterus, whether it can or cannot be differentiated from the right tube, it is wise to consider the possibility of the presence of an extra-uterine pregnancy.

In male patients the pain or distress of chronic prostatitis may be referred to the region of the appendix and chronic appendicitis simulated. It is well, therefore, to make a rectal examination in all cases of suspected chronic appendicitis in male patients. In fact, one may say that a rectal or vaginal examination should be made as a routine thing in the examination of all abdominal cases.

In like manner to the above, chronic chest lesions, may, in having pain referred to the abdomen, simulate appendicitis.

The value of X-ray investigation by a competent observer cannot be over estimated in the study of these cases. By it the position of the cæcum and its mobility or lack of mobility can be shown, and in some cases the actual appendix and its position outlined. It will also determine the presence or absence of obstruction at the ileo-cæcal valve. As an illustration of the importance of an exhaustive X-ray investigation we give a summary of the case of Mrs. D. of Montreal: The patient had been a more or less chronic invalid from gastro-intestinal disturbance for a period of ten years. The most prominent symptom had been gaseous distension with all its discomforts. Being a woman of means she consulted various eminent internists, both in this country and abroad. Various diagnoses were made and various treatments instituted. During that period she had two or three acute attacks of abdominal pain; these did not last long and were regarded as attacks of intestinal colic. In December, 1912, she was referred by her physicians to Dr. Pirie of Montreal for an X-ray investigation of her abdomen. This investigation, as will be seen in the attached diagrammatic report (see pages 555 to 557), lasted over a period of about six weeks. He found that soon after the taking of a meal containing barium sulphate, the appendix was outlined and that it continued to be outlined throughout the course of the investigation. He gave it as his opinion that the patient was suffering from chronic appendicitis. Her physicians, however, did not consider the retention of the barium in the appendix of any clinical significance. Soon after this time she was referred to Dr. Allen A. Jones of Buffalo for diagnosis. Dr. Pirie's report was regarded as important and it was decided to remove the appendix after a careful study had revealed nothing of importance to account for the symptoms. During the investigation in Buffalo abdominal massage with special reference to the right iliac fossa had been employed; whether this was provocative or not, an acute attack of appendicitis arose. Operation was performed a few hours after the onset of the attack and revealed an acute gangrenous appendicitis, from which the patient eventually made an excellent recovery.

As further illustrations of the importance of X-ray investigation we give a report of Dr. A. W. Bayliss and a series of reproductions from plates by Dr. Leonard Reu.

DR. A. W. BAYLISS' REPORT OF THE CASE OF MR. LAND.

"After making X-ray examinations of Mr. Land at different times, I am led to conclude that he has chronic appendicitis with a ptosis of the colon. Thinking his an operative case, I am referring him to you for your opinion and, if you deem it necessary, for operation."

The attached reproductions are from plates by Dr. Leonard Reu, and are taken from a series of plates in the investigation of each case (see pages 558 to 562):

Case A, figure 1, shows the appendix arising from the posterior surface of the cæcum, running outwards to the right, bending back upon itself and passing behind terminates at the inner side of the cæcum.

Case B, figure 2, shows a long, slender appendix passing upwards and inwards in a tortuous course.

Case C, figure 3, shows that the bismuth in the appendix has become broken up into segments.

Case D, figure 4, shows the appendix to be angulated near its extremity.

Case E, figures 5, 6 and 7, shows the retention of the bismuth throughout a period of 465 hours.

Case F, figures 8 and 9, shows obstruction at the ileo-cæcal valve. This case refused operative treatment in Buffalo; a few months later, however, he was operated upon in New York for the relief of intestinal obstruction.

We are convinced from our experience that the retention of bismuth or barium by the appendix is of great pathologic significance, for we may take it with a reasonable amount of certainty that an appendix which becomes filled with bismuth during an X-ray investigation is in constant danger of becoming filled with fecal matter; the fecal material as long as it remains liquid or semi-liquid is not likely to cause more than a catarrhal appendicitis, but if it be retained and converted into fecal concretions, abrasions of the mucous membrane may occur and form the port of entry for any bacteria that may be present within the lumen of the appendix. The inflammation arising from an invasion of the deeper tissues of the appendix by bacteria may vary from a mild one to a gangrenous appendicitis.

### The Treatment of Acute Appendicitis.

In discussing the treatment of acute appendicitis we shall deal with it in a general way, and shall not enter into any of the surgical problems met with during the operations for the relief of the disease, or into the complications which may occur during the course of it. The disease is essentially a surgical one and operation is demanded in a large percentage of the cases, and advisable in all cases, if not at the time of the acute attack at some subsequent date.

The question of the time of operation has been widely discussed. Many surgeons consider that the operation should be performed as soon as the diagnosis is made, irrespective of the duration of the attack. We are convinced, however, that no such sweeping statement holds in the treatment of acute appendicitis, and that each case must be considered on its own merits. As we have already pointed out in this discussion, there is a tendency to localization of the infection by the formation of a barrier of adhesions; this protecting barrier is one of the most important factors to be considered in the treatment of the disease. In the earlier stages, before localization has occurred, the safe procedure, the diagnosis being established, is to remove the appendix. During the third and fourth days the decision is a trying one to determine; if there be signs of localization it is probably preferable to be conservative and await the formation of an abscess; if, however, the signs point to a progression of the disease and infection of the general peritoneal cavity, the removal of the infecting cause and drainage of the peritoneal cavity is demanded. After the fourth day one may take it as a more or less general rule that it is wise to pursue a course of masterly inactivity and await the formation of an abscess. In so doing we are convinced that more patients will be saved than lost.

Later on in the history of the case when the abscess has formed and becomes well shut off from the general peritoneal cavity, it should be carefully opened at its most prominent and dependent point and drained. If the appendix presents itself it may be removed, but it is not wise to make any extensive search for it, on account of the danger of breaking down the limiting wall of adhesions. If, during the convalescence, a fecal fistula forms, one need not be alarmed, as it will close usually of its own accord. We have come to consider that the forming of a fistula in these cases is a blessing in disguise, as it shows that the appendix has become disintegrated and will give no further trouble. It should be a rule to remove the appendix at a later operation in all cases, in which a fecal fistula does not form, as it is a continual source of danger to the patient.

Acute appendicitis occuring during pregnancy is, as we have stated above, a serious complication to the pregnancy, but operation, if deemed necessary, should not be delayed on that account. It is wise to be as gentle as possible, as traumatism may excite an emptying of the uterus. It is also wise to administer morphine both before and after the operation to lessen reflex stimulation of the uterine muscles.

The use of the laboratory is as important during the treatment as in the diagnosis, and we make it a rule to investigate the appendix removed and any pus, if present. At the end of twenty-four hours the character of the infection is known and cultures are ready for the making of a vaccine, which is always used when indicated. The determination of the infecting cause is also of importance in helping us in the making of a prognosis.

It is a well established anatomical fact that the lymphatic papillæ are much less numerous in the lower than in the upper abdomen, and that as a consequence lymphatic absorption is at a minimum in the pelvis. In all acute abdominal conditions it is wise to recognize this and place the patient in such a position that intra-abdominal drainage is directed towards the pelvis. Fowler's position fulfills the requirements in this respect exceedingly well. Instillation of salt solution per rectum by the Murphy drop method is very valuable, because free absorption of the normal saline from the colon into the general circulation takes place; this allays thirst, dilutes the toxins in the general circulation, lessens the damage to the kidneys in their excretion of the toxins and bacteria, possibly increases peritoneal secretion and dilutes the products of the inflammation. The combination of the Fowler's position and the rectal instillation of normal saline by the Murphy method is an exceedingly valuable one in the treatment of acute abdominal infections, irrespective of the question of operation. Where it has not been employed prior to an operation is should be instituted immediately afterwards.

The role of morphine in the treatment of acute appendicitis has been very much discussed and much can be said for and against its use. We are convinced that it is under proper management invaluable in certain stages of the disease. By masking signs and clouding the clinical picture it is a most dangerous drug to employ before the diagnosis is established and the line of procedure for the treatment is determined upon. If operation is decided upon it may be used to relieve pain and to tranquillize the patient; immediately following the operation the same holds good. Where at the operation general peritonitis is encountered, or even feared, morphine should be pushed to the physiological limit, in order to thoroughly splint the intestines, and in the worst cases to completely stop peristalsis in order to lessen absorption and localize the infection. The same holds good in those cases which are seen late, and in which it is definitely decided not to operate, but to await the localization of the infection and the formation of an abscess or abscesses; in these latter cases it is wise to put the patient on a water diet, apply ice to the affected side, and make no attempt to move the bowels; however, the long rectal tube may be employed to tap the gases in the large bowel. Any attempt to move the bowels only increases the danger of spreading the infection.

In the treatment of chronic appendicitis the removal of the appendix is the one important thing to be considered. The operation should not be unduly delayed on account of the danger of a serious acute attack arising at any time. In those cases in which there are extensive adhesions causing obstruction at the ileo-cæcal valve, or elsewhere, it may be necessary to short circuit the ileum into the large bowel after the manner of Lane.

448 Delaware Ave.

---

After-History of Gastroenterostomy for Peptic Ulcer. Bourne (*British Medical Journal*) mentions the after-history of cases in which gastroenterostomy had been performed for ulcer of the stomach or duodenum. In the more recent cases there was an interval of at least 19 months between the operations and the inquiry as to the present conditions of the patient, while in the older patients more than five years had elapsed. Of the 92 cases of the series 68 have been traced. Of these, three have died in the interval. The total mortality of the operation, plus that of the uncured ulcer after operation, is 10.9 per cent. As to the results, nearly one-half the cases were in no way bettered. The operation was markedly successful in 43 per cent. and hopelessly bad in 38 per cent. The older the patient the better the outlook. The figures show that the result of operation depends very largely on the time after food at which pain occurs. This varies from immediately after the meal to one and a half hours, and in proportion as this interval is long, so is the prognosis good. Cases of duodenal ulcer as a rule do much better after this operation than cases of gastric ulcer. The degree of hyperchlorhydria is of importance, cases showing a total acidity of 0.2 to 0.3 per cent. reacting better than do those of a lesser percentage (0.12 to 0.19).

It will be noted that the mortality rate is high and the recovery rate low, as compared with the medical treatment but this may be because only severe and obstinate cases are operated upon. Why gastro-enterostomy should be employed for duodenal ulcer is scarcely apparent; if to relieve gastric acidity, other simpler means may be employed. 0.2 per cent. of HCl means about 50 degrees by the titration method, using decinormal alkali.

---

Mycosis Fungoides. C. M. Cole of Caldwell, Idaho, *Northwest Med.*, December, 1913, reports a fatal case, treated with X-ray and hypodermatics of sodium cacodylate.

---

Ophthalmic Tuberculin Reaction. Jacob Gutman of New York, *Arch. of Diag.*, October, 1913, discusses this test and concludes that no danger has been observed, that it is as reliable as the von Pirquet test, and that it should not be abandoned.

## Figures and Tables

**Figure f1:**
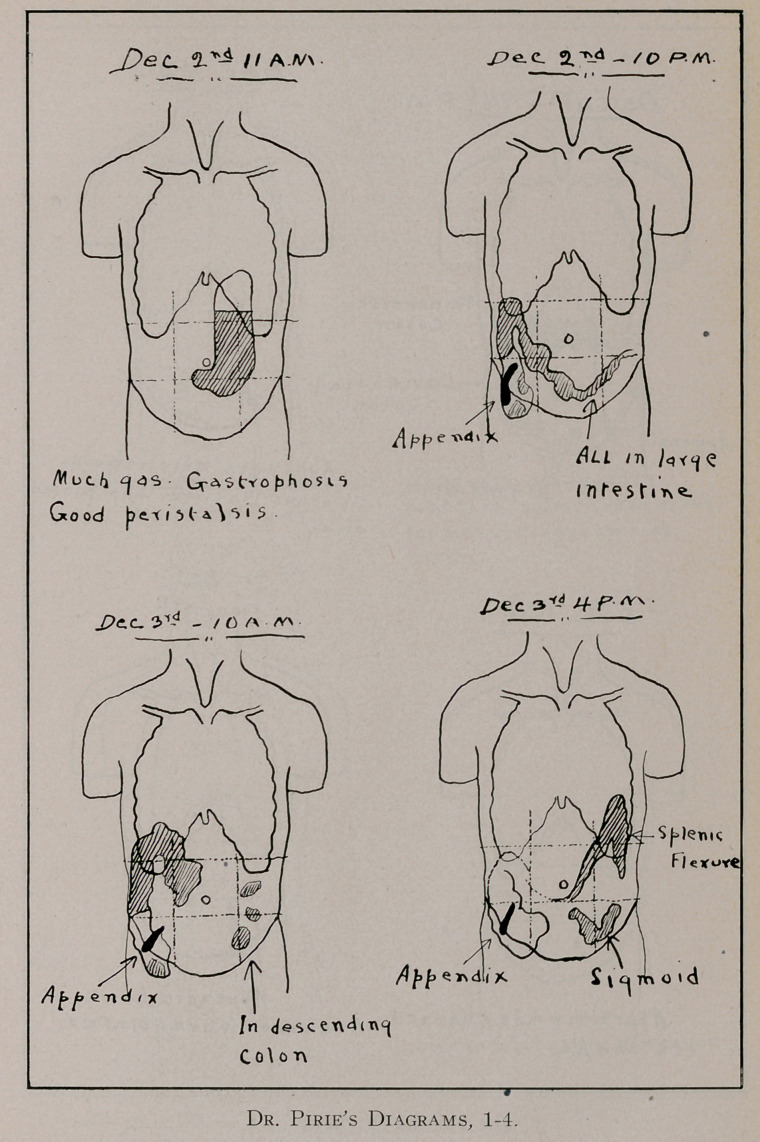


**Figure f2:**
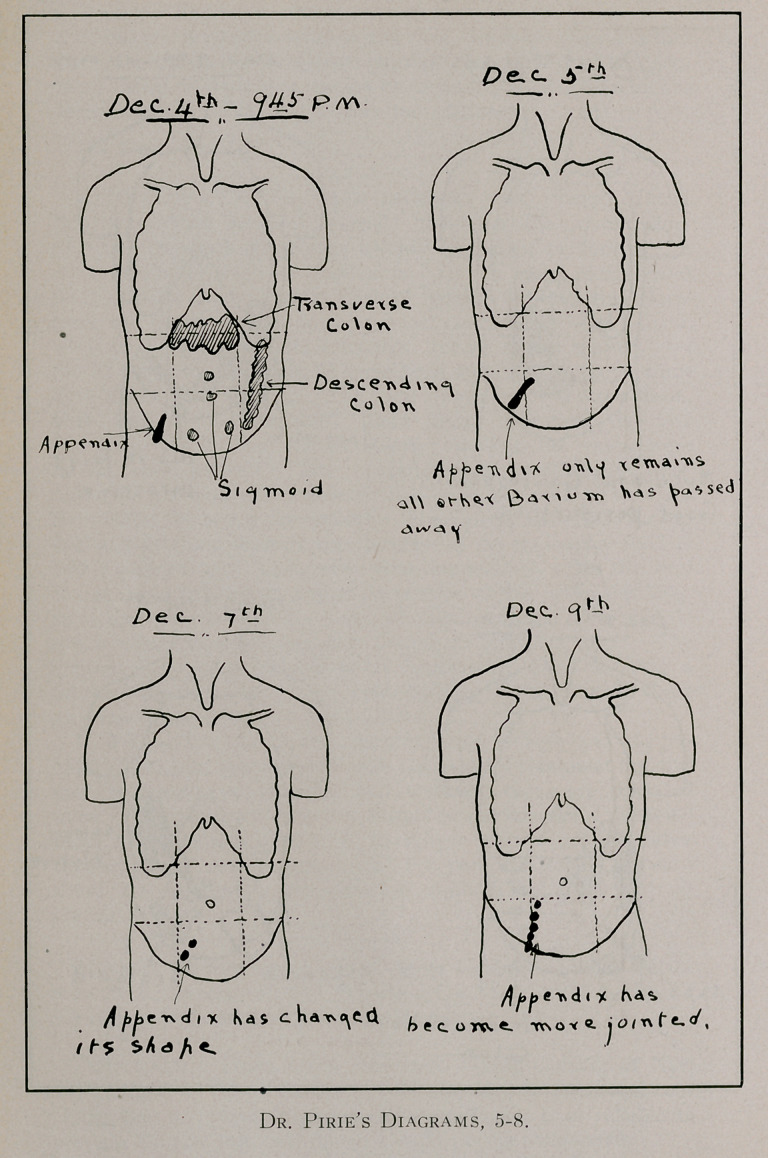


**Figure f3:**
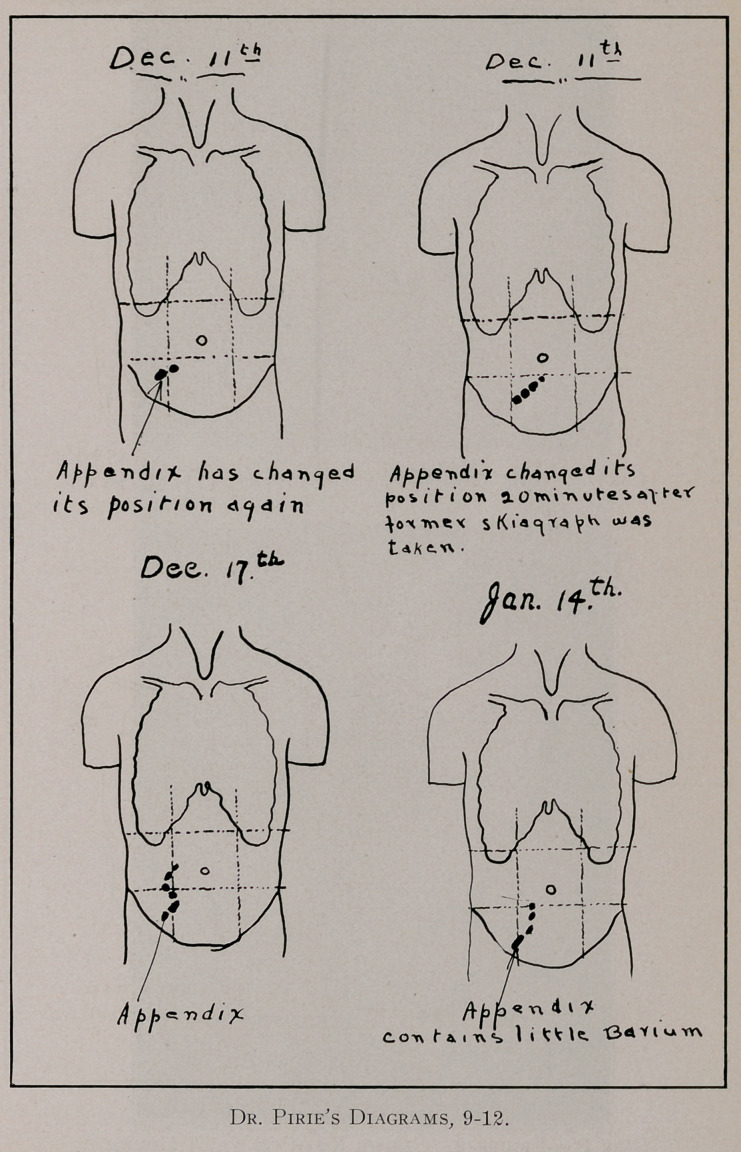


**Figure 1. f4:**
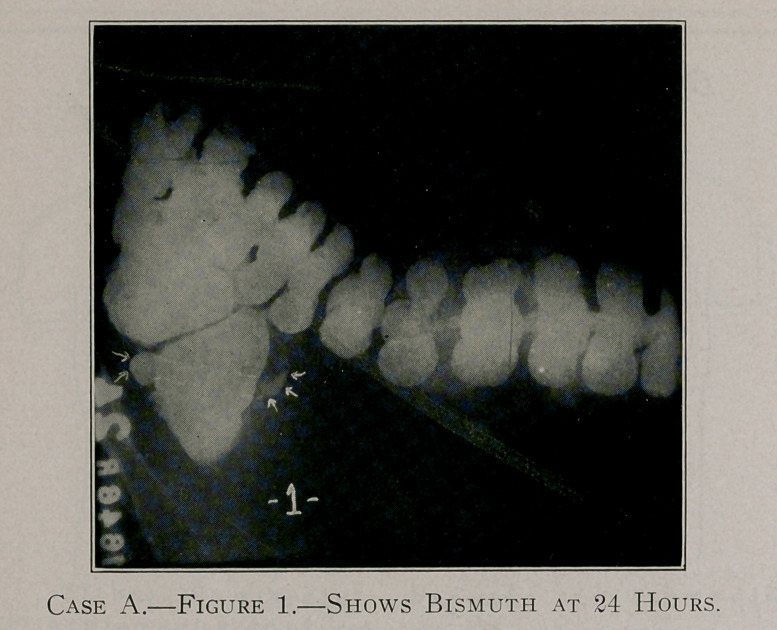


**Figure 2. f5:**
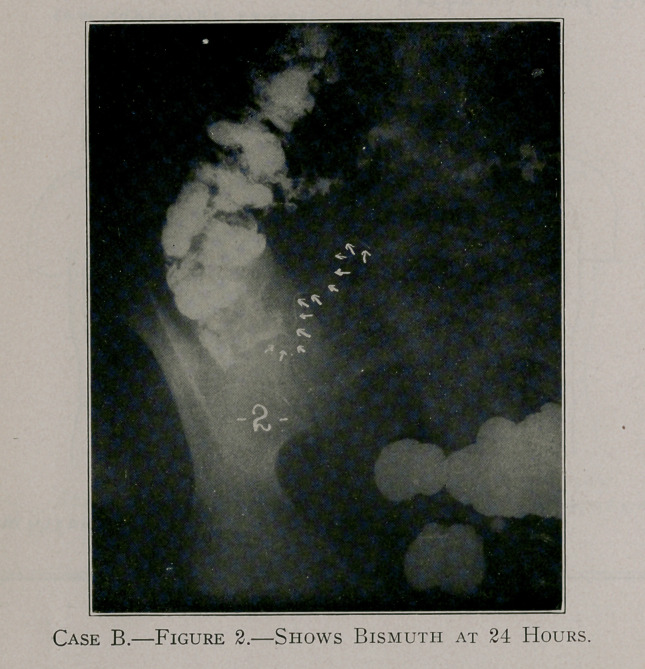


**Figure 3. f6:**
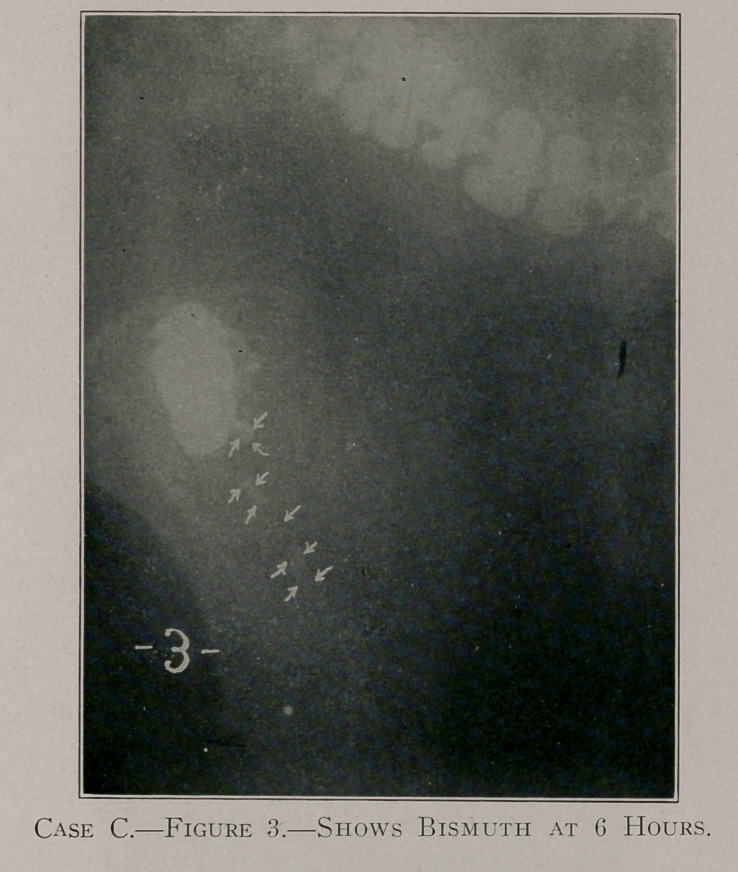


**Figure 4. f7:**
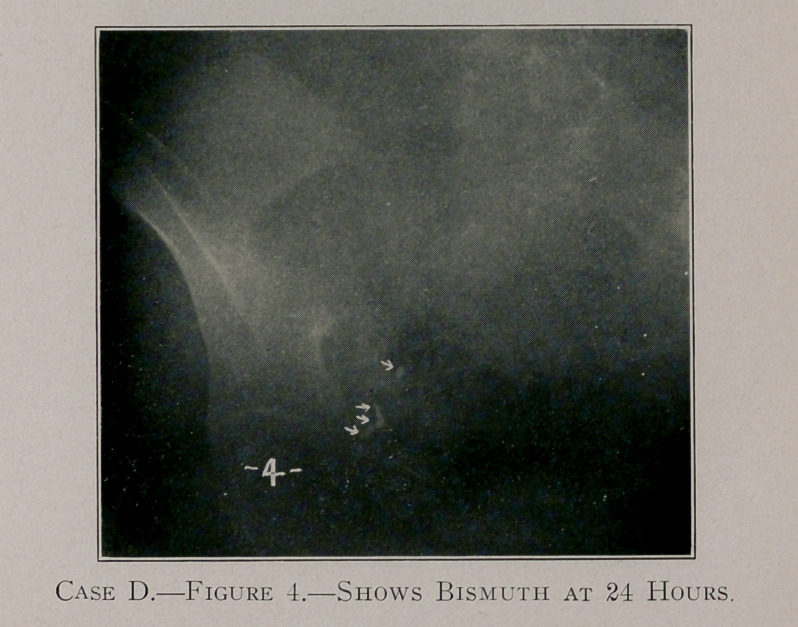


**Figure 5. f8:**
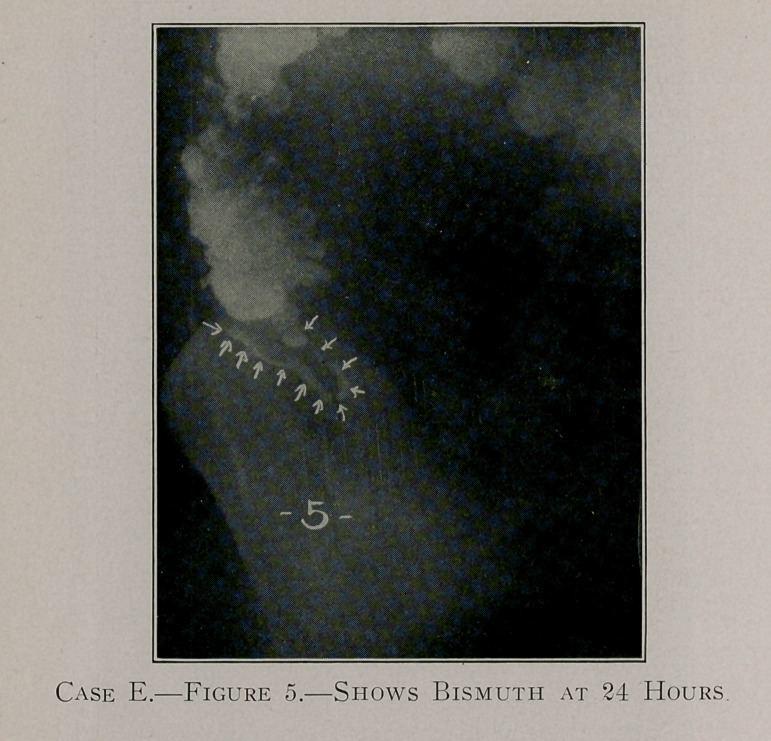


**Figure 6. f9:**
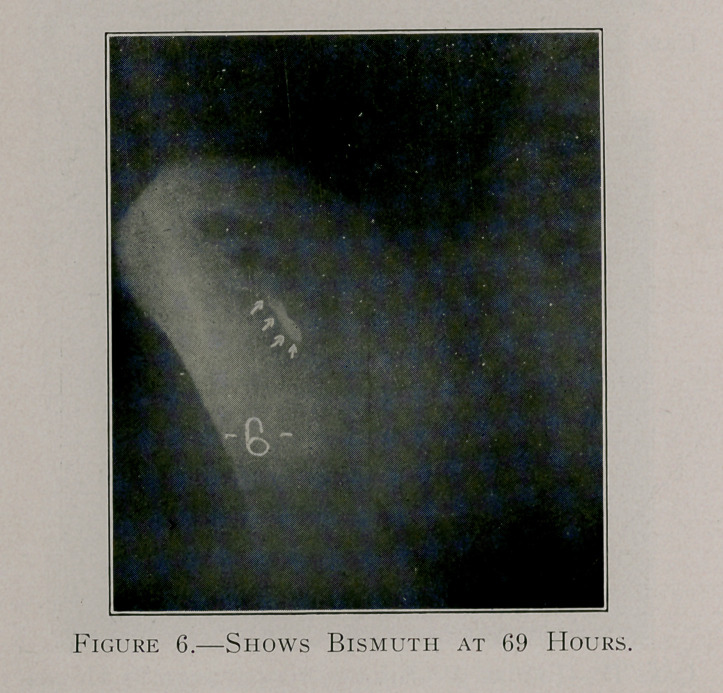


**Figure 7. f10:**
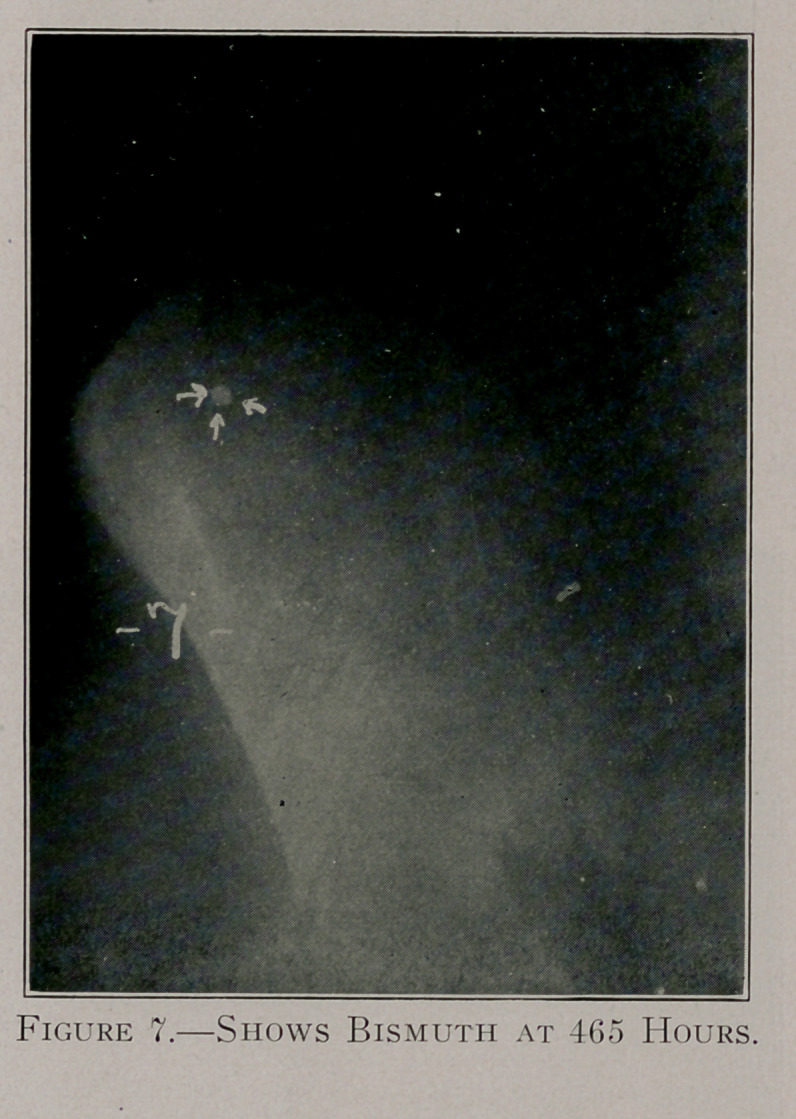


**Figure 8. f11:**
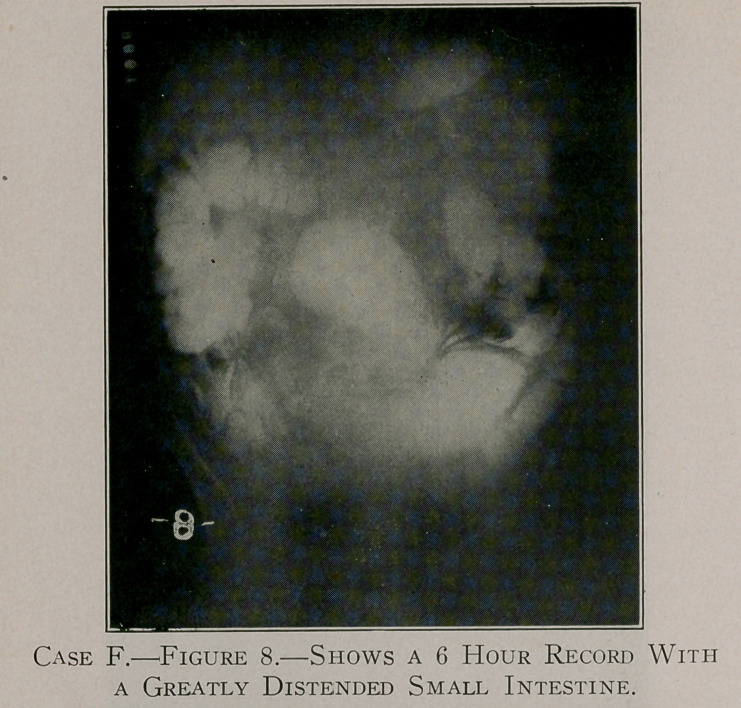


**Figure 9. f12:**